# Non-alcoholic fatty liver disease and diabetes

**DOI:** 10.1016/j.metabol.2016.01.001

**Published:** 2016-08

**Authors:** Jonathan M. Hazlehurst, Conor Woods, Thomas Marjot, Jeremy F. Cobbold, Jeremy W. Tomlinson

**Affiliations:** a Oxford Centre for Diabetes, Endocrinology and Metabolism, NIHR Oxford Biomedical Research Centre, University of Oxford, Churchill Hospital, Oxford, UK, OX3 7LE; b Department of Gastroenterology, Oxford University Hospitals NHS Trust, Oxford, UK, OX3 9DU

**Keywords:** NAFLD, NASH, Diabetes, Insulin resistance, Diabetes complications

## Abstract

Non-alcoholic fatty liver disease (NAFLD) and type 2 diabetes (T2DM) are common conditions that regularly co-exist and can act synergistically to drive adverse outcomes. The presence of both NAFLD and T2DM increases the likelihood of the development of complications of diabetes (including both macro- and micro- vascular complications) as well as augmenting the risk of more severe NAFLD, including cirrhosis, hepatocellular carcinoma and death.

The mainstay of NAFLD management is currently to reduce modifiable metabolic risk. Achieving good glycaemic control and optimising weight loss are pivotal to restricting disease progression. Once cirrhosis has developed, it is necessary to screen for complications and minimise the risk of hepatic decompensation.

Therapeutic disease modifying options for patients with NAFLD are currently limited. When diabetes and NAFLD co-exist, there are published data that can help inform the clinician as to the most appropriate oral hypoglycaemic agent or injectable therapy that may improve NAFLD, however most of these data are drawn from observations in retrospective series and there is a paucity of well-designed randomised double blind placebo controlled studies with gold-standard end-points. Furthermore, given the heterogeneity of inclusion criteria and primary outcomes, as well as duration of follow-up, it is difficult to draw robust conclusions that are applicable across the entire spectrum of NAFLD and diabetes. In this review, we have summarised and critically evaluated the available data, with the aim of helping to inform the reader as to the most pertinent issues when managing patients with co-existent NAFLD and T2DM.

## 1 Introduction

### 1.1 NAFLD is Common Among Individuals with Type 2 Diabetes

The prevalence of NAFLD varies widely depending on the population studied and the methodology applied. Studies have shown that NAFLD may be present in up to 70% of patients with diabetes [1], [2] whilst the prevalence of biopsy proven NASH (non-alcoholic steatohepatitis) in asymptomatic type 2 diabetics with normal liver function tests (LFTs) was 20% [3]. Estimates from our own studies and others have suggested that there is a significant burden of advanced fibrosis in asymptomatic individuals with type 2 diabetes ranging from 5% to 7% [4], [5]. There is therefore no doubt that these two common conditions co-exist and that there is significant amount of unrecognised advanced NAFLD within asymptomatic diabetic patients. Obesity and physical inactivity are interlinked risk factors for the development of diabetes and both are clearly implicated in an individual's risk of developing NAFLD. In a large cross-sectional study an individual's sitting time was associated with NAFLD diagnosed using US and interestingly this association held true in those with a normal BMI [6]. Obesity is well known to correlate with both NAFLD prevalence and severity. In a study of patients who had liver biopsies whilst undergoing elective abdominal surgery the BMI was strongly correlated with NASH [7] and in a separate study intraabdominal fat was associated with NASH [8].

### 1.2 NAFLD Increases Diabetes Risk, But the Reciprocal Relationship is Less Clear Cut

There is a strong association between NAFLD and diabetes risk. An individual's risk of developing diabetes is increased approximately 5-fold if they have NAFLD, although this is dependent on the population studied, duration of follow-up and methodology used to diagnose NAFLD [9], [10], [11], [12], [13], [14], [15], [16], [17], [18], [19], [20], [21], [22], [23] (Table 1). There is a considerable degree of heterogeneity among these studies and in one of the longest prospective studies, the observed increased risk for developing type 2 diabetes (19% vs 6% for non-NAFLD) was found to be non-significant after adjusting for confounding variables. However, the diagnosis of NAFLD at baseline was made on the basis of abnormal LFTs without imaging and it is therefore likely that some individuals classified as non-NAFLD may have had a degree of hepatic steatosis or even more advanced disease [24]. Importantly, improving NAFLD has been shown to modify the risk of developing diabetes [25]. Currently, we are not able to predict which individuals with NAFLD will develop diabetes and annual surveillance of HbA1c is likely to be the most pragmatic solution although some data suggest that an OGTT may be more accurate in the context of NAFLD reflecting post-prandial glucose excursions [26], [27].

Whether type 2 diabetes increases an individual's risk of developing NAFLD is less clear cut and harder to study. A large proportion of patients with type 2 diabetes are diagnosed long after the onset of their diabetes which means that it is difficult to design studies assessing the duration of diabetes and the risk of developing NAFLD although common sense would dictate that there is a positive association. Given the insidious nature of type 2 diabetes, it is not surprising that those with established diabetes have markedly more liver fat when compared to age, BMI and gender matched controls [28]. However in cross-sectional analysis of 99,969 apparently healthy, non-diabetic Korean individuals, there was an increased risk of NAFLD (as determined by USS (ultrasound scan)) with increasing levels of HbA1c and insulin resistance, independent of obesity [29]. This introduces the concept of ‘pre-diabetes’ as a possible precursor for NAFLD and its subsequent progression. A small cross-sectional series of non-diabetic individuals found that impaired glucose tolerance (IGT) or impaired fasting glucose (IFG) alone occurred in 25% of patients with simple steatosis versus 55% of those with NASH [30]. In a study of 108 patients with serial liver biopsies (median interval 6.6 years) at baseline those with NASH rather than NAFL were more likely to be diabetic (56% vs 21%) and importantly those who had advanced fibrosis or cirrhosis at follow up biopsy were more likely to be diabetic at follow up than those without advanced fibrosis (89% vs 47%) [31]. Taken together these findings highlight the importance of diabetes to NAFLD disease progression.

Whilst insulin resistance is implicated in NAFLD pathogenesis there is still continued debate as to whether this represents a cause or consequence [32]. Conversely, some studies have shown that NAFLD risk may actually be lower in patients with type 1 diabetes in comparison with controls, however in these studies, differences in visceral adipose, lipid profiles and LFTs in the control cohorts as well as the high prevalence of NAFLD in overweight asymptomatic individuals may mask any increased risk [33], [34]. However, these observations are supported by a paediatric study where the control individuals and patients had similar lipid profiles, although it should be noted that the differences in hepatic lipid percentage was small [35].

### 1.3 NAFLD Increases Risk of Diabetes Complications and Diabetes May Increase the Risk of NAFLD Progression

NAFLD (diagnosed on ultrasound and excluding other causes of liver disease) increases the risk of cardiovascular events by 1.87-fold of an individual with type 2 diabetes after adjusting for confounders [36]. Although a separate study did not identify increased mortality, in this retrospective analysis the cohort investigated was composed of those who underwent CT scanning for a specific clinical indication and this may have had an additive effect on mortality risk, potentially masking any impact of NAFLD [37]. It is important to recognise that neither of these studies used liver biopsies and as a consequence was not able to differentiate between NAFLD and NASH which may be relevant to cardiovascular disease risk [38]. As well as cardiovascular risk [36], co-existent NAFLD increases the risk of microvascular complications of diabetes including chronic kidney disease and retinopathy [39]. Furthermore, hepatic fat content has been shown to be associated with increased insulin requirements [40] which have the potential to fuel weight gain. The available data linking NAFLD to diabetes complications are limited in that they are mostly taken either retrospectively or from observational cohort studies rather than from longitudinal data.

There is emerging evidence demonstrating an additive detrimental liver outcome for people with co-existent diabetes and NAFLD. A diagnosis of diabetes makes an individual more likely to have more severe NAFLD with the associated complications of cirrhosis and mortality. In one large cohort study, the standardised mortality ratio from cirrhosis was increased in diabetics (2.52) [41]. Furthermore, in a series of 432 patients with biopsy proven NAFLD the presence of co-existent type 2 diabetes was found to be an independent risk factor for fibrosis [42]. Other smaller studies that included liver biopsies have identified an additive effect of NAFLD and diabetes on cirrhosis, liver and all-cause mortality [43]. In another study, those patients with periportal–portal fibrosis were more likely to have diabetes [44]. In studies using serial biopsies those with progressive fibrosis were more likely to be diabetic at baseline and were also more likely to develop diabetes if not already diagnosed [31], [45]. Finally, in a meta-analysis, co-existent diabetes was associated with a poorer prognosis in individuals with hepatocellular carcinoma [46]. Overall therefore, the evidence seems clear that co-existent NAFLD and diabetes are associated with a more severe adverse outcome than either of the conditions in isolation.

### 1.4 Pathophysiology

The multi-hit pathogenesis of NAFLD has been reviewed extensively [47], [48], [49] and is briefly outlined below. In addition, bile acids and the gut microbiota are now believed to have a crucial role in the pathogenesis of NAFLD and their putative contribution has also been reviewed elsewhere [50], [51].

Insulin resistance within the liver as well as extra-hepatic tissues such as adipose and skeletal muscle is implicated in the pathogenesis of NAFLD, however there are emerging data describing hepatic steatosis occurring in the absence of insulin resistance, particularly in individuals with single nucleotide polymorphisms within the *PNPLA3* gene that encodes the enzyme, patatin-like phospholipase 3 [52]. The accumulation of triacylglycerol (TAG) within the liver comes from three sources: 59% from circulating free fatty acids (FFAs); 26% from *de novo* lipogenesis (DNL); and 14% from the diet [53]. FFAs entering the portal circulation undergo one of 3 fates: β-oxidation; re-esterification to TAG and export as VLDL (very low-density lipoprotein); or re-esterification and storage within the liver. DNL, the process whereby carbohydrates are converted to lipid, also contributes to lipid accumulation within the liver. DNL is increased in states of hyperinsulinaemia such as insulin resistance [54]. Gluconeogenesis, the generation of glucose from non-carbohydrate sources, is also increased in individuals with NAFLD [55]. In addition to providing a substrate for DNL, increases in intrahepatic glucose and the glycolytic product, pyruvate, increase the production of acetyl-CoA and increase the proportion of acetyl-CoA converted to malonyl-CoA for DNL, rather than allowing it to enter the citric acid cycle [56]. All of the above mechanisms contribute to the development of hepatic steatosis. A relatively small percentage (≈ 23%) of those with simple steatosis progress to steatohepatitis [57]. The precise contributions of the multifactorial causes of this inflammatory switch are less clear, but are of importance as the presence of steatohepatitis is associated with the development of progressive disease and of poorer outcomes in some series [58]. Oxidative stress [59], mitochondrial dysfunction [55] and circulating cytokines [60] have all been implicated in the transition from simple steatosis to NASH which may then progress to fibrosis. Finally, an additional ‘hit’ has been proposed that contributes to the failure of hepatocytes to regenerate promoting further fibrosis.

### 1.5 Emerging and Existing Treatments

The remaining part of this review will focus on emerging treatments as well as the application of existing diabetes drugs in the treatment of NAFLD. It is important to note that the studies examining the treatment effect in NAFLD are heterogeneous, relating to variability in inclusion criteria (simple steatosis through to NASH and cirrhosis) as well as primary outcomes that range from normalisation of ALT or improvement on MRS (magnetic resonance spectroscopy) of hepatic fat through to histological improvement. There are no medications currently licenced for the treatment of NAFLD. The mainstay of treatment remains addressing metabolic risk factors with particular attention to weight loss via life style interventions. Caloric restriction and exercise are proven to improve liver histology. Even a relatively short period of caloric restriction (28 days) has been shown to markedly improve liver steatosis in a cohort of highly motivated living liver lobe donors whose initial biopsies showed they were not suitable to donate [61]. High intensity training 30–40 min a week for 12 weeks has been shown to reduce hepatic steatosis measured with MRS [62]. In a large series of patients who had liver biopsies pre and post 52 weeks of advice on caloric restriction and exercise the degree of weight loss achieved was strongly correlated with histological improvement. In this study 25% of patients had resolution of NASH and 19% had regression of fibrosis [63].

### 1.6 Beyond Insulin Resistance: Insulin and Liver Fat

Whilst insulin resistance and the associated hyperinsulinaemia are detrimental to the liver, there are data showing that the exogenous administration of insulin to type 2 diabetic patients can be beneficial [64]. 12 weeks of insulin glargine therapy and not the comparator Liraglutide when administrated to patients with type 2 diabetes inadequately controlled on oral agents resulted in reduced hepatic fat as measured by MRS [64]. This finding corroborates observations from previous studies [65], [66]. Although insulin promotes lipogenesis and decreases lipid oxidation [67] *in vitro*, the paradoxical improvement in liver fat seen in human studies [64], [65], [66] may be mediated by increased TAG secretion [68] or improved hepatic insulin sensitivity and reduced gluconeogenesis [66]. Insulin remains the most efficacious drug to optimise glycaemic control in patients with diabetes and is comparatively safe across the stages of NAFLD. In contrast, there are little data on the safety of many oral hypoglycaemic agents in severe liver disease and concerns about weight gain and the use of insulin therapy should not override its use when needed for glycaemic control. However, there are some retrospective data associating insulin treatment with fibrosis [69] and hepatocellular carcinoma [70] and these are discussed in more detail below.

## 2 The Role of Existing Oral Hypoglycaemic Agents

### 2.1 Metformin, a Common Sense Rather Than Evidence-Based Approach

Metformin is currently the first line therapeutic agent in the management of patients with type 2 diabetes. Whilst the improvements in HbA1c are modest, it has been shown to lower body fat and improve hepatic insulin sensitivity when measured by suppression of hepatic gluconeogenesis during a hyperinsulinaemic clamp [71]. *In vitro*, the activation of AP-activated protein kinase by metformin results in increased fatty acid oxidation and reduced DNL [72], [73], but these observations have not translated to reduced hepatic steatosis in human studies [71]. In a large meta-analysis that included 671 individuals (27% of whom were diabetic), despite improvements in HbA1c and weight, there was no statistically significant histological improvement in hepatic steatosis or inflammation [74]. Currently, there are no longitudinal data examining the effect of metformin on mortality in NAFLD. The use of metformin in severe liver disease remains controversial with emerging evidence suggesting that it may be safe in cirrhosis and even improve mortality. In a retrospective analysis, patients with type 2 diabetes who continued metformin at diagnosis of cirrhosis (Child's Pugh A) and in particular NASH cirrhosis, had a markedly longer median survival than those who discontinue metformin [75]. These data are interesting and whilst only 3 patients had metformin discontinued because of elevated serum creatinine, it is important to note that in the multivariate analysis, the data were not adjusted for creatinine, which may have improved the survival data in the metformin group. In this study, no individuals developed lactic acidosis. Furthermore, *in vitro* data as well as population studies suggest that metformin may have a beneficial effect in reducing hepatocellular carcinoma (HCC) risk [76]. A retrospective study looking at metformin use and HCC mortality found no benefit [77], but in a separate retrospective study in diabetic patients with HCC, those on metformin that underwent radiofrequency ablation had a reduced mortality compared to those not on metformin after adjustment for confounders including tumour size and glycaemic control [78]. In conclusion, metformin is not licenced for use in NAFLD outside the context of diabetes. Although there is no discernible improvement in steatosis or histological features of NASH, there are data suggesting improved survival in patients with cirrhosis and hepatocellular carcinoma and it is at this end of the NAFLD spectrum where the underlying mortality burden is increased.

### 2.2 Sulphonylureas are Associated With Weight Gain, but Remain an Effective Treatment for Glycaemic Control

Sulphonylureas (SU) are commonly used as second line agents for glycaemic control in patients with type 2 diabetes. They act upon the SUR1 subunit of the inward-rectifying potassium channel of the β-cells of the pancreas, causing the channel to close, which leads to cellular depolarisation, resulting in the opening of voltage gated Ca^2 +^ channels and consequent insulin release. There are no prospective data examining their use in NAFLD with co-existent diabetes. Some retrospective data exist suggesting that the prevalence of fibrosis in diabetic patients with NAFLD is higher in individuals treated with sulphonylureas [69]. However no adjustment was made for glycaemic control or diabetes duration. The authors proposed a profibrotic effect of insulin as a potential mechanism. *In vitro* experiments have described a profibrotic effect of insulin increasing the proliferation of hepatic stellate cells and the accumulation of type 1 collagen [79]. In a combined analysis of studies comparing oral hypoglycaemic agents taken for 1 year, gliclazide treatment was associated with a modest deterioration of LFTs when used either as a single agent or in combination [80]. These modest changes in LFTs are likely to be distinct from the rare cases reported of gliclazide-induced hepatitis [81].

In a large meta-analysis, an association between HCC and SU or insulin use was identified, however subsequent *post-hoc* analysis did not reveal any significant association between diabetes therapy and HCC [70]. This may yet be important particularly in the context of previously published work showing that those exposed to SUs or insulin have an increased risk of mortality [82], although the authors acknowledge that missing data (including smoking status and glycaemic control) may well confound these results. Given the availability of generic SUs it is unlikely that prospective studies will address the outstanding issues that surround their use in the context of NAFLD, however as this class of agent is associated with a gain in weight and is metabolised extensively by the liver, it is unlikely to be an attractive treatment option for diabetic patients with NAFLD.

### 2.3 Thiazolidinediones are Effective at Reducing Liver Fat but Safety Concerns Persist

Thiazolidinediones (TZDs) are insulin sensitising agents that are selective ligands of the peroxisome-proliferator-activated receptor ϒ (PPARϒ). PPARs are important transcription factors implicated in metabolic homeostasis. They are particularly effective at sensitising adipose tissue to insulin, promoting fatty acid uptake and storage [83] and this may be the pre-dominant mechanism by which they improve hepatic steatosis [84] particularly given that PPARϒ expression within the liver is increased in individuals with NAFLD [85]. Different isoforms of PPAR have been implicated in the fate of fatty acids with PPARϒ particularly important in re-esterification, PPARα in β-oxidation and PPARβ/δ in gluconeogenesis. The relevant contributions of these isoforms are therefore likely to be pivotal in the pathogenesis of NAFLD [86]. PPARβ/δ agonists are currently in active development, whilst fibrates which activate PPARα are widely prescribed as lipid lowering agents. The use of fibrates in NAFLD is outside of the context of this review which is focussed specifically on the diabetes and NAFLD, however, no discernible histological or clinical benefit has been shown with fibrate therapy [87] and animal data suggest that they may in fact promote hepatic steatosis [88].

TZDs are highly selective agonist for PPARϒ. Animal studies using TZDs in models of NAFLD have been of limited value as full agonism of PPARϒ can promote hepatic lipogenesis, which contrasts with the findings seen in human studies. Most of the early data with TZDs and NAFLD were taken from studies examining the use of Rosiglitazone, which has now been withdrawn from use in Europe and is restricted in the USA because of an association with cardiovascular events. Pioglitazone is still prescribed, but its use has been limited by concerns regarding adverse effects including fracture risk and an association with bladder cancer [89], [90]. In the FLIRT trial, 63 participants with biopsy confirmed NASH were randomised to receive either rosiglitazone or placebo for 1 year. Whilst steatosis improved, there was no improvement in fibrosis or in the NAFLD activity score [91]. In the open-label extension, there was no additional benefit [92]. In a study of 55 individuals with either diabetes or impaired glucose tolerance who were treated for 6 months with either pioglitazone or placebo, there were improvements in steatosis, ballooning and inflammation but not fibrosis [93]. A 12-month study of pioglitazone in non-diabetics also demonstrated some histological improvement [94]. In the PIVENS study of 247 non-diabetics with NASH Pioglitazone was compared to Vitamin E and to placebo over 96 weeks. The primary outcome was a composite end-point that included improvement in the NAFLD activity score as well as improvements in ballooning score with no deterioration in fibrosis. Given the three treatment arms, a *p*-value of < 0.025 was required for significance and whilst Pioglitazone did not achieve significance for the primary outcome, there were improvements in many of the secondary histological outcomes including the NAFLD activity score [95]. The effect of pioglitazone may be mediated by increases in adiponectin which is known to have effects on the liver that include reducing gluconeogenesis and reducing fatty acid influx [96]. Despite encouraging data, concerns regarding fluid retention, weight gain and to a lesser extent bladder cancer have meant that the use of pioglitazone in diabetic patients with NAFLD remains limited. TZDs are not licenced for the treatment of NAFLD in non-diabetic individuals.

### 2.4 The Efficacy of DPP-IV Inhibitors in NAFLD is Uncertain

Dipeptidyl peptidase IV (DPP-IV) is a cell surface peptidase than in addition to inactivating the incretins such as GLP-1, is known to have a role in modulating T cell immune response. DPP-IV inhibitors increase the availability of incretins which are responsible for incretin effect whereby the insulin response to glucose is greater to an oral rather than IV glucose challenge. Serum DDP-IV is increased in individuals with NASH compared to controls and serum and liver staining for DPP-IV within individuals with NASH correlates to histopathological grade [97]. More recently the same relationship has been demonstrated for DDP-IV serum activity in a large cohort [98].

DPP-IV inhibitors are now widely prescribed as adjunctive oral therapy in patients with type 2 diabetes. DDP-IV inhibitors improve insulin sensitivity and hepatic steatosis in animal models of diet-induced obesity with some evidence of improved liver inflammation [99] and may also limit progression to fibrosis in animal models of liver injury [100]. To date, there are no robust data with histological end-points as a primary outcome to formally comment on the effectiveness of DPP-IV as a treatment for NAFLD with co-existent diabetes. However, a small retrospective study has shown DPP-IV inhibitors to be a safe and efficacious treatment for glycaemic control in those patients with diabetes and NAFLD [101]. In a small, non-randomised study of individuals with ultrasonagraphic steatosis, DDP-IV inhibition was associated with improved glycaemic control and reduced AST and ALT [102] and in a prospective blinded randomised controlled study, 6 months of treatment reduced hepatic triglyceride as measured by MRS [103]. The evidence relating to lipid profile changes with DPP-IV inhibition is more conflicting [103], [104], with some evidence to suggest a post prandial benefit [104].

Currently, there is insufficient evidence to be able to discriminate between the different DDP-IV inhibitors that are available to be prescribed to treat co-existent NAFLD and diabetes. Prescribing advice remains to use caution in more severe hepatic impairment although this class of agents is predominantly renally excreted [105].

### 2.5 GLP-1 Agonists are a Promising Treatment for NAFLD and Larger Phase III Studies are Warranted

Glucagon-like peptide-1 (GLP1) is released from L-cells of the small intestines in response to nutrients passing through the small intestine. GLP-1 is an insulin mimetic but also has many important extra-pancreatic effects including on satiety and increasing insulin sensitivity [106]. In addition to glycaemic control GLP-1 analogues are an effective treatment for obesity including in non-diabetic individuals [107] and have been granted a licence for use as weight loss therapy by both FDA and EMA.

GLP-1 analogue therapy improves hepatic steatosis [108] and steatohepatitis [109] in ob/ob mice as well as wild type mice fed an obesogenic diet [109]. It is not clear whether the mechanism that underpins the improvement in the hepatic phenotype is exclusively due to weight loss. Rodent studies with weight loss matched controls as well as GLP-1 receptor knock-out animals suggest that effects are not entirely mediated by weight loss alone, but are dependent upon on the expression of the GLP-1 receptor [109]. Furthermore, GLP-1 agonists have a direct action to suppress lipogenesis in rodent hepatocytes [110].

The presence of GLP-1 receptors on human hepatocytes is controversial with conflicting data [111], [112]. Whilst the underpinning mechanisms remain to be fully elucidated in humans, the GLP-1 agonist Liraglutide has been shown to be safe and well tolerated in patients with type 2 diabetes and its use is associated with a reduction in ALT [113]. A 12-week intervention with Liraglutide did not improve liver fat on MRS [64], however, although currently only published in abstract form, Liraglutide has been shown to be an effective treatment for those with NASH both with and without diabetes [114] in a placebo controlled study with improvement in liver biopsy at 52 weeks as the primary outcome. Furthermore, in a study incorporating hyperinsulinaemic–euglycaemic clamps, Liraglutide has been shown to improve hepatic and adipose insulin sensitivity [115].

### 2.6 SGLT2 Inhibitors May Offer Promise Although Future Studies in NAFLD Will Have to Address Changes in Lipid Profile

SGLT2 inhibitors (sodium glucose cotransporter 2) are a new class of oral hypoglycaemic agents that work by decreasing renal glucose reabsorption. The net effect of increased renal glucose excretion serves the dual purpose of glycaemic control and calorie loss although there are concerns about changes to volume status and blood pressure [116]. However, animal models of NAFLD with SGLT2 inhibitors have demonstrated a protective effect on steatosis, inflammation and fibrosis [117], [118]. This attenuated steatosis–fibrosis progression may well be due to a combination of negative energy balance through glycosuria and substrate switching towards lipids as a source of energy expenditure [119]. There are currently no human studies of SLGT2 inhibitors and NAFLD, however, given the net weight loss of 1.8 kg seen in a published meta-analysis [120] it may represent an attractive strategy, but this remains to be investigated in dedicated clinical studies. It is important to note that this weight loss in humans is largely mediated by a reduction in fat mass rather than osmotic diuresis [121].

### 2.7 Bariatric Surgery in the Context of NAFLD and Diabetes

Bariatric surgery is an effective treatment for obesity and has been shown to markedly improve and even cure diabetes [122] as well as improve histological features in NAFLD [123]. Much of this is likely mediated by weight loss, although it is thought that an improved incretin effect contributes to improvements in glycaemic control [124]. The improvements in histology are often accompanied by improved glycaemic control [125] but there are no robust data to determine whether one is required for the other. In a retrospective analysis of 756 patients who underwent bariatric surgery improvement in ALT was associated with an improvement in diabetes with ALT remaining elevated in those remaining on insulin therapy [126].

## 3 Conclusions

Diabetes and NAFLD are reciprocal risk factors and when they are occur together, an increasing body of data demonstrates that diabetes is more difficult to manage and that NAFLD is more likely to progress. As NAFLD represents a spectrum from simple steatosis through to cirrhosis and is itself diagnosed by a variety of methods, it is no surprise that there is considerable heterogeneity within the epidemiological studies. Screening asymptomatic diabetic patients for NAFLD remains controversial and there are concerns both about the volume of unrecognised severe NAFLD as well as the management and health economic realities of making this diagnosis. Screening those with established NAFLD for diabetes is not controversial and should be undertaken.

The pathogenesis of NAFLD is complex although the accumulation of intrahepatic lipid is central. Whilst insulin resistance both in peripheral tissues and within the liver contributes to this, the evidence from studies looking at *PNPLA3* demonstrates that insulin resistance is not an absolute requirement for the development NAFLD. However, insulin resistance in adipose tissue and skeletal muscle remains an important component in the pathogenesis of NAFLD leading to increased circulating glucose and lipid substrate availability for hepatic lipid accumulation.

Studies examining the treatment effect of anti-diabetic medications are also extremely heterogeneous with current evidence suggesting only limited disease modifying effects across different classes of agent. Many of these studies have focussed on the accumulation of intrahepatic lipid measured either with ultrasound or more recently with MRS. Whilst reducing intrahepatic lipid is important and biologically relevant, studies examining future therapies may be better targeted to more severe NAFLD, including NASH with fibrosis and cirrhosis, as it is at this end of the disease spectrum where there are significant increases in both liver and cardiovascular mortality.

There are many challenges in the diagnosis and management of NAFLD in patients with diabetes. Tailoring an individual treatment strategy to optimise metabolic control with the potential to improve liver phenotype is the current gold-standard. Further research is needed to define the causative mechanism that drives NAFLD progression in patients with diabetes as well as assessing the impact of newer anti-diabetic treatments and identification of additional novel targets.

## 4 Future Directions

There are no currently licenced pharmacological agents specifically designed to treat NAFLD. Bariatric surgery remains an efficacious treatment for diabetes and obesity. In a recent meta-analysis weight loss ranged between 20% and 50% and rates of diabetes remission between 40% and 90% with the studies included in this analysis having a duration of between 12 and 24 months [127]. Given the metabolic benefits afforded by bariatric surgery it is not surprising that a second meta-analysis has shown marked improvements in liver histology including a 50% reduction in the incidence of steatosis and an 11.9% reduction in the incidence of fibrosis pre and post bariatric surgery [123]. Whilst bariatric surgery is undoubtedly effective there are limitations including complications, patient acceptability, service availability and cost [128]. Future medical therapies therefore may well be designed to artificially induce some of the changes seen following bariatric surgery. The two areas receiving most attention in this regard are bile acids and gut hormones. The bile acid profile is different in patients with biopsy proven NASH compared to controls with increased levels of bile acids dominated by taurine- and glycine-conjugated primary bile acids and secondary bile acids [129]. It should be noted in this study that control individuals were not matched for BMI, diabetes status or lipids all of which are likely to be relevant to bile acid metabolism. The effects of bile acids post weight loss surgery are far ranging and include mediating appetite, lipid metabolism, gut hormones, glucose homeostasis, energy metabolism, gut microbiota and endoplasmic reticular stress [130]. Consensus is that bile acid levels increase following Roux-en-Y gastric bypass (RYGB) in the fasting state although the effect on postprandial levels is more debatable [131]. Many of these effects are mediated via the Farsenoid X nuclear receptor. Obeticholic acid is a synthetic variant of the natural bile acid chenodeoxycholic acid and is a potent agonist of the FXR. In a double-blind placebo controlled study of individuals with non-cirrhotic NASH obeticholic acid treatment was associated with both resolution of NASH and improvements in fibrosis at 72 week liver biopsy [132]. Whilst these are encouraging data the long term safety features and efficacy need to be addressed, furthermore the efficacy in patients with poor glycaemic control will need to be established.

Gut hormones have been found to be increased after RYGB especially in the post-prandial state. Hormones studied include oxyntomodulin (OXM), GLP-1 and Peptide YY [133]. GLP-1 analogues or DPPIV inhibitors (thereby increasing the availability of endogenous GLP-1) are already licenced for use in type 2 diabetes and the relevant data regarding NAFLD are discussed above. It has been proposed that to best replicate the effects seen following RYGB pharmacologically future strategies may involve combinations of the relevant hormones [134].

## Conflict of Interest

Jonathan M Hazlehurst and Jeremy W Tomlinson have been involved in the LEAN study (Clinical trials.gov identifier NCT01237119) investigating Liraglutide in NASH however neither of them has received financial support from Novo Nordisk.

## Figures and Tables

**Table 1:** The risk of developing diabetes in individuals with NAFLD.

Reference	Country	Population	NAFLD assessment	Duration follow up	T2DM risk	Method for diagnosis of T2DM	Adjusted for
Shibata et al. 2007 [9]	Japan	3189^a^ -802 NAFLD -2387 normal	US	4	5.5 HR	FPG + OGTT	Age, BMI
Kim et al. 2008 [10]	Korea	5372 -1790 NAFLD -3582 normal	US	5	1.51 RR	Treatment, FPG, history	Age, gender, ETOH, smoking, BMI, TGs, HDL-c, FPG, ALT, USonographer
Adams et al. 2009 [11]	Australia	358 -109 NAFLD -249 normal	ALT	11	NS	FPG, self-reporting	
Balkau et al. 2010 [12]	France	863 -277 NAFLD -594 normal	FLI < 20 FLI ≥ 70	9	Men: 4.71 OR Women: 22.71 OR	FPG, treatment	Age, ETOH, glucose, insulin, physical activity, smoking, FH Diabetes, BP
Yamada et al. 2010 [13]	Japan	12,375 NAFLD/normal not reported	US	5	1.91 OR men 2.15 OR women	FPG	Age, BMI, BP, ETOH
Sung et al. 2011 [14]	Korea	11,091 -8120 NAFLD - 2971 normal	US	5	2.05 OR	FPG	Age, gender, BMI, ETOH, education, smoking, activity, FPG
Bae et al. 2011 [15]	Korea	8849 - 2292 NAFLD -5557 normal	US	4	1.33 HR	FPG, medications	Age, gender, BMI, TG, HDL-C, BP, IFG, smoking, activity, ETOH
Chon et al. 2012 [16]	Korea	1161^a^ -107 NAFLD -1054 normal	US	4	7.63 OR	FPG or HbA1c	Age, BMI
Sung 2012 [17]	Korea	12,853 -3555 NAFLD -9298 normal	US	5	2.42 OR	FPG	Age, gender, education, smoking, activity, ETOH, ALT, TGs
Choi JH 2013 [18]	Korea	7849 -NAFLD US 951, ALT 1147, both US and ALT 1341 -normal 4410	US ALT	5	ALT 1.2 HR (NS) US 1.03 (NS) Both ALT and US 1.64	FPG, HbA1c	Age, gender, activity, smoking, ETOH, BMI, TG, HDL-C, BP, IFG,
Kotronen et al. 2013 [19]	Finland	4512 -646 NAFLD -3866 normal	FLS	15	3.81 RR	treatment	Age, gender, BMI, smoking, activity, TGs, BP, HDL-c
Park et al. 2013 [20]	Korea	25,232^a^ mild NAFLD 7709, mod-severe 1149 − 16,374 normal	US	5	Mild 1.09 HR Mod-severe 1.73 HR	FPG, HbA1c	Age, WC, TGs, HDL-C, BP, CRP, HOMA-IR, creatinine, FH diabetes, Exercise, MetS (IDFTF)
Zelber-Sagi et al. 2013 [21]	Israel	141 -35 NAFLD -106 normal	US	7	2.93 OR	PreDM: FPG or HbA1c	Age, gender, BMI, FH diabetes, baseline insulin, adiponectin and glucose
Ming et al. 2015 [22]	China	508^a^ -97 NAFLD -411 normal	US	5	4.462 RR	OGTT	Age, gender, Education, ETOH, BMI, FH Diabetes, BP, fasting glucose, 2 h glucose, TG, LPL
Jager et al. 2015 [23]	Germany	487 -431 NAFLD -56 normal	FLI < 30 FLI ≥ 60	10	17.6 HR	Self reporting, medications, medical records	Age, Education, Activity of occupation, smoking, Activity of leisure, ETOH, Intake of coffee, red meat, whole-grain

NAFLD; normal = not NAFLD; US = ultrasound; FLI = fatty liver index; FLS = fatty liver score; HR = hazard ratio; OR = odds ration; T2DM = type 2 diabetes mellitus; FPG = fasting plasma glucose; OGTT = oral glucose tolerance test; BMI = body mass index; ETOH = alcohol consumption; TGs = triglycerides; HDL-c = high-density lipoprotein cholesterol; ALT = alanine transaminase; USonogapher = Sonographer performing the ultrasound; FH diabetes = family history of diabetes; BP = hypertension; IFG = impaired fasting glucose; HbA1c = glycosylated haemoglobin; MetS (IDDF) = metabolic syndrome as defined by IDDF; LPL = lipoprotein lipase.

a Only male participants.
